# Exploring changes in metabolites and fecal microbiota of advanced gastric cancer based on plasma metabolomics and 16S rDNA sequencing

**DOI:** 10.1016/j.heliyon.2025.e41715

**Published:** 2025-01-06

**Authors:** Xinyi Feng, Yu Zhang, Jun Feng, Zhongjun Li, Zhi Zhang, Lin Zhu, Ruoyu Zhou, Haibo Wang, Xiaojun Dai, Yanqing Liu

**Affiliations:** a Institute of Translational Medicine, Medical College, Yangzhou University, Yangzhou, 225001, China; b The Key Laboratory of Syndrome Differentiation and Treatment of Gastric Cancer of the State Administration of Traditional Chinese Medicine, Yangzhou, 225001, China; c Department of Oncology, Gaoyou Hospital of Traditional Chinese Medicine, Yangzhou 225600, China; d Department of Oncology, Yizheng Hospital of Traditional Chinese Medicine, Yangzhou 225600, China; e Department of Oncology, Baoying People's Hospital, Yangzhou 225600, China; f Department of Oncology, Yangzhou Hospital of Traditional Chinese Medicine, Yangzhou 225600, China

**Keywords:** Plasma metabolomics, 16S rDNA sequencing, Gastric cancer, Gut flora

## Abstract

Metabolomics and 16S rDNA sequencing have shown great potential in elucidating complex mechanisms associated with diseases. Currently, there is little research on the omics of gastric cancer and it lacks effective biomarkers.

**Objective:**

Based on plasma metabolomics and 16S rDNA sequencing to evaluate the changes in metabolites and fecal microbiota of advanced gastric cancer.

**Method:**

Firstly, plasma metabolomics was used to screen for differential metabolites and metabolic pathways in gastric cancer. Then, 16S rDNA sequencing was performed on fecal samples to study the differential intestinal microbiota in gastric cancer patients. Finally, conduct a correlation analysis between them.

**Result:**

A total of 152 differential metabolites were identified, and we screened 10 of them. All metabolites were enriched into 42 differential metabolic pathways, of which 13 have P values less than 0.05. 16S rDNA sequencing showed significant differences in 4 microbial communities at the phylum level. There are significant differences in 23 communities at the genus level. We focus on Lactobacillales, Lactobacillus, Streptococcus, Veillonella, Bacilli and Megasphaera. Correlation analysis shows that the intestinal microbiota and plasma metabolites jointly affect the occurrence and development of gastric cancer.

**Conclusion:**

For the first time, we comprehensively used plasma metabolomics and 16S rDNA sequencing to reveal the changes and correlations between metabolites and intestinal microbiota in advanced gastric cancer. We have discovered new potential biomarkers for gastric cancer. This deepens our understanding of the physiological and pathological mechanisms of advanced gastric cancer and helps to improve the diagnosis and treatment of advanced gastric cancer.

## 1 Introduction

Gastric cancer (GC) is a malignant tumor with high incidence rate and high mortality in China. At present, the treatment of advanced gastric cancer mainly relies on surgery, radiotherapy and chemotherapy, but its toxicity and side effects seriously affect the patients' quality of life [1,2]. And it is prone to recurrence and metastasis. This greatly affects the overall treatment effect of gastric cancer, and the prognosis of patients is extremely poor [3,4].

Gastric cancer is a highly heterogeneous disease driven by multiple genetic mutations and epigenetic abnormalities. It involves changes in multiple genes, pathways and stages [5]. Due to the lack of accurate and reliable biomarkers, the diagnosis and treatment of gastric cancer still have significant limitations. With the emergence of gene sequencing, biological analysis and big data analysis tools, finding its biomarkers is crucial for diagnosing diseases, predicting disease progression and treating diseases.

Currently, metabolomics and 16S rDNA sequencing are becoming powerful tools for discovering biomarkers and revealing pathological processes.

An increasing number of studies indicate that cancer cells promote survival and proliferation by altering multiple metabolic pathways. The reprogrammed cellular metabolism supports the occurrence and development of tumors [6,7]. We use metabolomics to detect cancer in order to discover its key biomarkers. Targeted regulation of metabolism is expected to become a powerful therapeutic strategy for cancer [8,9]. Metabolomics studies the endogenous metabolites of organisms and their relationship with influencing factors. It is a science about the types, quantities, and changes of metabolic substances in living organisms [10].

16S rDNA sequencing can detect microorganisms in specific environments, providing a variety of information such as sample species classification, species abundance and population structure. It is one of the most commonly used bacterial classification standards. In recent years, with the fast advances in high-throughput sequencing, the intestinal microbiota has gradually attracted people's attention. More and more evidence suggests that intestinal microbiota has local or remote regulatory functions on target organs. The balance of them is closely related to human health [11,12]. Disrupting their balance will lead to a series of diseases. Studying the diversity and composition of intestinal microbiota in gastric cancer patients provides a theoretical basis for disease research and treatment [13].

This study aims to analyze the differences in plasma metabolites and intestinal microbiota between gastric cancer patients and the normal population, in order to discover new targeted biomarkers for gastric cancer.

## 2 Materials and methods

### 2.1 Research object

Referring to the diagnostic criteria for gastric cancer in the "Diagnosis and Treatment Guidelines for Common Malignant Tumors in China", the participants were 30 patients with stage IIIB-IV advanced gastric cancer who visited Yangzhou Traditional Chinese Medicine Hospital, Gaoyou Traditional Chinese Medicine Hospital, Yizheng Traditional Chinese Medicine Hospital and Baoying Traditional Chinese Medicine Hospital from July 2022 to December 2022 (referring to the staging criteria of the American Joint Commission on Cancer (AJCC) 8th edition). The study has been approved by the Ethics Committee of Yangzhou Traditional Chinese Medicine Hospital in Jiangsu Province((2022) Lun Shen No. (31)).

#### 2.1.1 Inclusion criteria

(1) Age range from 18 to 75 years old, regardless of gender.
(2) Gastric cancer patients with clear pathological diagnosis.
(3) Gastric cancer patients with stages IIIB-IV.
(4) The expected survival period is more than 3 months.
(5) KPS score ≥60 points.
(6) Participants have good compliance and must sign an informed consent.

#### 2.1.2 Exclusion criteria

(1) Individuals who have experienced significant traumatic damage such as surgical treatment in the past month.
(2) Individuals with severe bleeding or uncontrolled infection.
(3) Individuals with persistent purulent and chronic infected wounds.
(4) Individuals with uncontrolled diseases such as splenic hyperfunction, hyperthyroidism, connective tissue disease, and tuberculosis.
(5) Patients with severe cardiovascular and cerebrovascular complications, active hepatitis, and severe liver and kidney dysfunction.
(6) People who take drugs that affect the intestinal microbiota, such as microbial regulators and antibiotics.
(7) Individuals suffering from malabsorption syndrome and other diseases that affect gastrointestinal absorption, as well as eating difficulties.
(8) Individuals with or experiencing malignant tumors in other parts of the body (excluding fully treated cervical carcinoma in situ, skin basal or squamous cell carcinoma, or other tumors that have been cured surgically and have not recurred for at least 5 years).
(9) Women of childbearing age who have tested positive for urinary pregnancy, are breastfeeding, or have insufficient contraception.
(10) There are any unstable or potentially hazardous situations that may endanger patient safety and affect compliance with the study, such as those with severe mental illnesses such as schizophrenia.
(11) There are any circumstances that may affect the test results, such as someone having eaten before being sampled for blood.

### 2.2 Metabolomics

#### 2.2.1 Chemicals and reagents

All of the standards of targeted metabolites were obtained from Sigma-Aldrich (St. Louis, MO, USA), Steraloids Inc. (Newport, RI, USA) and TRC Chemicals (Toronto, ON, Canada). All the standards were accurately weighed and prepared in water, methanol, sodium hydroxide solution, or hydrochloric acid solution to obtain individual stock solution at a concentration of 5.0 mg/mL. Appropriate amount of each stock solution was mixed to create stock calibration solutions. Formic acid was of (Optima grade and obtained from Sigma-Aldrich (St. Louis, MO, USA). Methanol (Optima LC-MS), acetonitrile (Optima LC-MS), and isopropanol (Optima LC-MS) were purchased from Thermo-Fisher Scientific (FairLawn, NJ, USA). Ultrapure water was produced by a Mill-Q Reference system equipped with a LC-MS Pak filter (Millipore, Billerica, MA, USA).

#### 2.2.2 Sample preparation

Samples were thawed on ice-bath to diminish sample degradation. 20 μL of plasma was added to a 96-well plate. Then the plate was transferred to the Eppendorf epMotion Workstation(Eppendorf Inc., Humburg, Germany). 120 μL ice cold methanol with partial internal standards was automatically added to each sample and vortexed vigorously for 5 min. The plate was centrifuged at 4000g for 30 min (Allegra X-15R, Beckman Coulter, Inc., Indianapolis, IN, USA). Then the plate was returned back to the workstation. 30 μL of supernatant was transferred to a clean 96-well plate, and 20 μL of freshly prepared derivative reagents was added to each well. The plate was sealed and the derivatization was carried out at 30 °C for 60 min. After derivatization, 330 μL of ice-cold 50 % methanol solution was added to dilute the sample. Then the plate was stored at −20 °C for 20 min and followed by 4000g centrifugation at 4 °C for 30 min 135 μL of supernatant was transferred to a new 96-well plate with 10 μL internal standards in each well. Serial dilutions of derivatized stock standards were added to the left wells. Finally the plate was sealed for LC-MS analysis.

#### 2.2.3 Instrumentation

An ultra-performance liquid chromatography coupled to tandem mass spectrometry (UPLC-MS/MS) system (ACQUITY UPLC-Xevo TQ-S, Waters Corp., Milford, MA, USA) was used to quantitate a l l t a r g e t e d metabolite s in this project. The optimized instrument settings are briefly described below. The instrument performance optimization and routine maintenance were performed every week. (Table 1, The optimized instrument settings)

**Table 1:** The optimized instrument settings.

UPLC-MS/MS instrument settings	
UPLC	
Column	ACQUITY UPLC BEH C18 1.7 μM VanGuard pre-column (2.1 × 5 mm) and ACQUITY UPLC BEH C18 1.7 μM analytical column (2.1 × 100 mm)
Column Temp. (°C)	40
Sample Manager Temp. (°C)	10
Mobile Phases	A = water with 0.1 % formic acid; and B = acetonitrile/IPA (70:30)
Gradient Conditions	0–1 min (5 % B), 1–11min (5–78 % B), 11–13.5 min (78–95 % B), 13.5–14 min (95–100 % B), 14–16 min (100 % B),16–16.1 min (100-5% B),16.1–18 min (5 % B).
Flow Rate (mL/min)	0.40
Injection Vol. (μl)	5.0
MASS SPECTROMETER	
Capillary (Kv)	1.5 (ESI+), 2.0 (ESI-)
Source Temp (°C)	150
Desolvation Temp (°C)	550
Desolvation Gas Flow (L/Hr)	1000

#### 2.2.4 Data analysis

##### 2.2.4.1 Software

The raw data files generated by UPLC-MS/MS were processed using the MassLynx software (v4.1, Waters, Milford, MA, USA) to perform peak integration, calibration, and quantitation for each metabolite. The iMAP platform (v1.0; MetaboProfile, Shanghai, China) was used for statistical analyses.

##### 2.2.4.2 Quantitation

Mass spectrometry-based quantitative metabolomics refers to the determination of the concentration of a substance in an unknown sample by comparing the unknown to a set of standard samples of known concentration (i.e., calibration curve). The calibration curve is a plot of how the analytical signal changes with the concentration of the analyte (the substance to be measured). For most analyses a plot of instrument response vs. concentration will show a linear relationship. This yields a model described by the equation y = ax + b, where y is the instrument response e.g., peak height or area, a represents the slope/sensitivity, and b is a constant that describes the background. The analyte concentration (x) of unknown samples may be calculated

from this equation.

##### 2.2.4.3 Multivariate statistical analyses

Partial Least Square Discriminant Analysis (OPLS-DA) algorithm decomposes the raw data set into three parts: systemic variations, orthogonal/unrelated information, and residual. This leads to a model with a minimal number of predictive components defined by the number of degrees of freedom (k − 1 dimensions) between group variances (k classes). This partitioning of the X-data facilitates model interpretation and model prediction.

### 2.3 16SrDNA sequencing

Stool samples from all participants were freshly collected and frozen at −80 °C within 3 h after sampling.

#### 2.3.1 DNA extraction, PCR amplification and sequencing

Microbial DNA was extracted from fecal samples using an E.Z.N.A.® soil DNA Kit (Omega Bio-tek, Norcross, GA), according to the manufacturer ‘s protocols. The NanoDrop 2000 UV–vis spectrophotometer (Thermo Scientific, Wilmington, Delaware, United States) was used to determine the final DNA concentration and purity, and DNA quality was checked using 1 % agarose gel electrophoresis. The hypervariable regions of the bacterial 16S rRNA gene were amplified with the primers by a thermocycler PCR system (GeneAmp 9700, ABI, Foster, CA, United States). The PCR reactions were as follows: denaturation at 95 °C for 3 min, 27 cycles of denaturation at 95 °C for 30s, annealing at 55 °C for 30s, elongation at 72 °C for 45 s, extension at 72 °C for 10 min, and ending at 4 °C. Each 20 μL reaction mixture contained 4 μL of 5 × TransStart FastPfu buffer, 2 μL of 2.5 mM dNTPs, 0.8μ L of each primer (5μ M), 0.4μ L TransStart FastPfu DNA Polymerase, and 10 ng template DNA. PCR was performed in triplicate. The PCR product was extracted from 2 % agarose gel and purified using an AxyPrep DNA Gel Extraction Kit (Axygen Biosciences, Union City, CA), according to the manufacturer's instructions, and quantified using Qubit 4 (Thermo Fisher, United States). Purified amplicons were pooled in equimolar and paired-end sequenced on the Illumina MiSeq PE300 platform (Illumina, San Diego, United States) according to the standard protocols by Honsunbio Technology Co. Ltd (Shanghai, China).

#### 2.3.2 Bioinformatics analysis

Sequencing reads were demultiplexed, quality controlled by fastp (version 0.21.0), and merged by FLASH (version 1.2.7). Shortly, reads with adaptor sequences and low quality bases (quality score < Q20) were trimmed. Truncated reads shorter than 50 bp and reads containing ambiguous nucleotides were discarded. Subsequently, the paired-end reads were merged according to the minimum overlap of 10 bp with maximum mismatch ratio of 0.2 in the overlapping region. Only merged sequences were retained for downstream analyses. The UPARSE algorithm was used to cluster sequences with a 97 % similarity cutoff, while chimeric sequences were identified and removed. Next, the taxonomy of each OTU representative sequence was assigned by using RDP Classifier against the reference database SILVA138 with a minimum confidence score of 0.7. Rarefaction was performed in order to compare the abundance of OTUs across samples. Sequences demultiplexed were imported to QIIME2 (version 2022.8). The DADA2 algorithm was used to quality filter and denoise sequences, while chimeric sequences were removed. Next, the taxonomy of each ASV representative sequence was assigned by using RDP Classifier against the reference database SILVA138 with a minimum confidence score of 0.7. The number of sequences from each sample was normalized to the lowest number of read counts by randomly selecting subsets of sequences.

#### 2.3.3 Statistical analysis

The R (version 4.1.3) was used to perform general statistical analysis and visualize results via packages vegan (v2.6–4), phyloseq (v1.38.0), tidyverse (v1.3.2), ggpubr (v0.5.0), ComplexHeatmap (v2.10.0) and corrplot (v0.92). Alpha diversity was estimated using the Sobs, ACE, Chao1, Shannon and Simpson indices. Principal coordinates analysis (PCoA) based on bray-curtis matrices with statistical significance determined by permutational multivariate analysis of variance (PERMANOVA) was conducted to assess the differences in beta diversity between groups. For comparing the relative abundance of different taxa between groups, linear discriminant analysis (LDA) effect size (LEfSe) method was performed with a p-value <0.05 for the Kruskal–Wallis test and a size-effect threshold of 2.0 on the logarithmic LDA score. Spearman's rank correlation analysis was used for correlation analysis.

## 3 Results

### 3.1 Metabolomics

Metabolomics technology has successfully discovered a variety of cancer-related metabolite markers, which can reveal the differences in metabolites under pathological conditions and provide new insights for early diagnosis and treatment of diseases. Studies have pointed out that amino acid transport in many malignant tumor cells is significantly increased. For example, the metabolism of gastric cancer often requires a large consumption of serine and glutamine [14]. Metabolites that have been identified for distinguishing gastric cancer include isoleucine, lactic acid, glutamic acid, glutathione, etc. Their expression levels change with the progression of gastric cancer. Metabolic pathways such as glycolysis, tricarboxylic acid cycle, and glutamine catabolism in gastric cancer are also affected to varying degrees [15]. Different syndrome types of the disease have different levels of energy metabolism and lipid metabolism. For example, He Nana et al. discovered 10 related biomarkers for gastric cancer with spleen deficiency and dampness excess [16]. Currently, the study of gastric cancer with other syndrome type has also become a new research hotspot.

#### 3.1.1 Metabolite classification

**Fig. 1:**
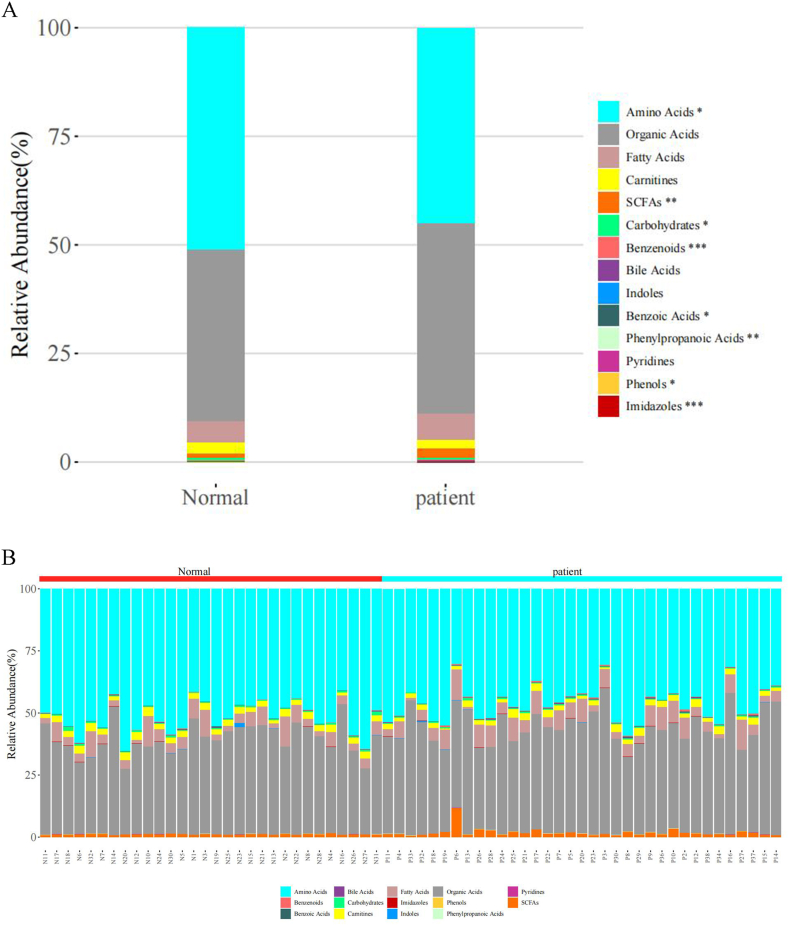
(A) Stacked bar chart showing the relative abundance of the median values of various metabolite types in each sample group; (B) Stacked bar chart displaying the relative abundance of various metabolite types in each individual sample.

We conducted an in-depth analysis of the metabolites present in all samples and detected 14 different categories of metabolites. Compared to the plasma metabolites in the normal group, the expression of 8 metabolites in gastric cancer exhibits significant differences.These metabolites include benzenoids, imidazoles (p < 0.001), short-chain fatty acids, phenylpropanoic acids (p < 0.01), phenols, amino acids, benzoic acids, and carbohydrates (p < 0.05). Among them, the absolute value of the log2FC of benzenoids is 1.71. Notably, except for the decreased expression of amino acids in the gastric cancer patient group, the expression of other categories of metabolites has increased (see Fig. 1A and B).

#### 3.1.2 Identification of differential metabolites

**Fig. 2:**
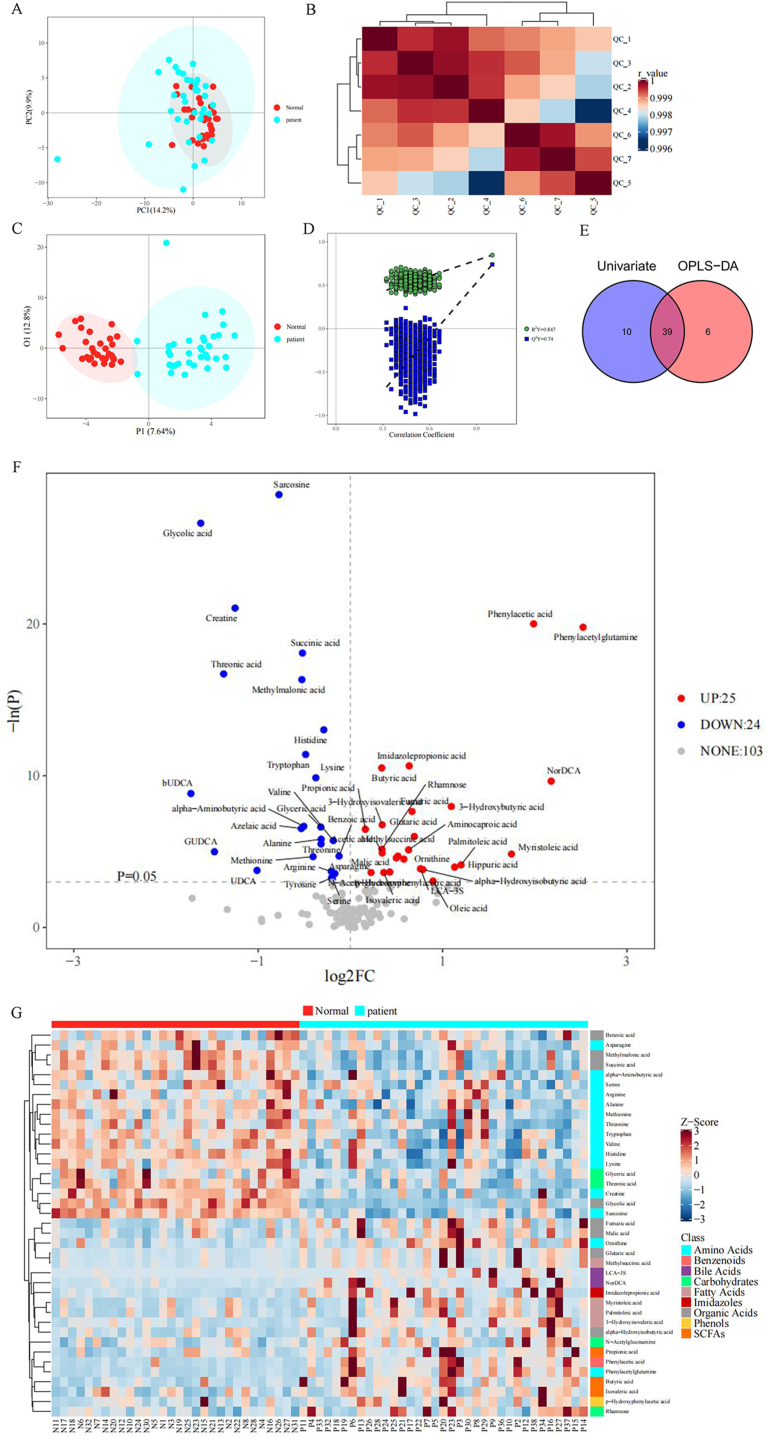

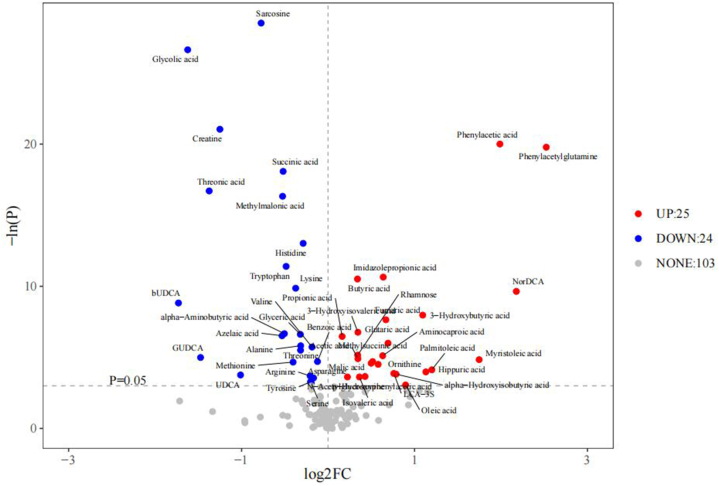
(A) PCA 2D score plot; (B) Heatmap of correlations between QC samples; (C) OPLS-DA 2D score plot; (D) OPLS-DA permutation test results; (E) Venn diagram of differentially expressed metabolites screened by multidimensional and unidimensional methods; (F) Volcano plot of unidimensional metabolites; (G) Heatmap of potential biomarkers.

First, we performed a multidimensional statistical analysis. The results of the principal component analysis showed that the contribution rate of the first principal component was 14.2 %, and the contribution rate of the second principal component was 9.9 % (Fig. 2A). The proximity of QC sample points in the PCA score plot indicates good stability of the instrument detection. In the heatmap of correlations between QC samples (Fig. 2B), the color of each cell represents the correlation coefficient between a QC sample and other QC samples. A correlation coefficient closer to 1 indicates that the QC samples are more similar to each other. This indicates that the experimental detection data is more stable and the quality control is effective. Then, we utilized Orthogonal Partial Least Squares-Discriminant Analysis (OPLS-DA) for analysis. The horizontal coordinate P1 represents the first predictive principal component of the model, and the vertical coordinate O1 represents the first orthogonal component of the model. The percentages in parentheses represent the explanatory power of the principal components, and the points of different colors represent different sample groups (Fig. 2C). To prevent overfitting of the OPLS-DA model, we set a threshold with Q2Y > 0.2 in the OPLS-DA model, and the intercept of the Q2Y fitting curve on the Y-axis in the permutation test was less than 0. The permutation test results showed that R2Y = 0.847 and Q2Y = 0.74. This indicates that the model has good stability and predictive performance. (Fig. 2D).

Based on the normality and homogeneity of variance of the data, we further conducted a one-dimensional statistical analysis. The volcano plot of one-dimensional metabolites shows the fold change and p-value of each metabolite screened for differential metabolites (Fig. 2F). The threshold for selecting differential metabolites is: (1) P < 0.05; (2) |log2FC| ≥ 0. The vertical dashed line represents the log-transformed FC threshold, with log2FC as the x-coordinate. The horizontal dashed line represents the P = 0.05 threshold, with the corresponding -logeP value as the y-coordinate. Points that satisfy both being above the horizontal dashed line and on both sides of the vertical dashed line will be highlighted. The red highlights on the right representing metabolites with increased concentration in the gastric cancer group. The blue highlights on the left representing metabolites with decreased concentration in the gastric cancer group. Gray points represent metabolites that do not meet the set threshold requirements. The results showed that compared with the healthy group, there were 152 differentially expressed plasma metabolites, of which 25 metabolites were upregulated and 24 metabolites were downregulated (Fig. 2F).

After taking the intersection of the differentially expressed metabolites obtained from the aforementioned unidimensional and multidimensional statistical analyses (unidimensional test p < 0.05, multidimensional statistics VIP>1), we selected the biomarkers relevant to this study for subsequent analysis (Fig. 2E). A total of 55 differentially expressed metabolites were identified, with 6 metabolites only present in the multidimensional results, 10 metabolites only in the unidimensional results, and 39 metabolites are common to both (Fig. 2E). The heatmap of potential biomarkers shows that these metabolites mainly including amino acids, benzene ring compounds, bile acids, carbohydrates, fatty acids, imidazoles, organic acids, phenolic compounds, and short-chain fatty acids (Fig. 2G).

#### 3.1.3 Screening of characteristic diagnostic metabolites based on random forest algorithm and ROC curve analysis

**Fig. 3:**
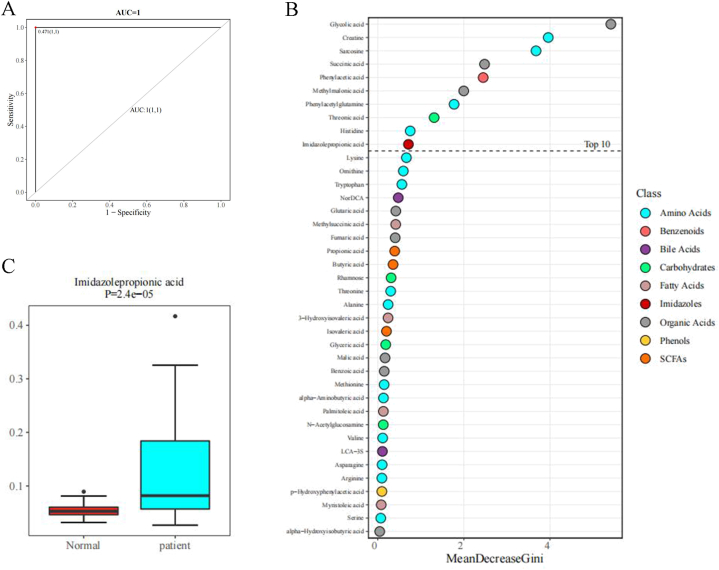

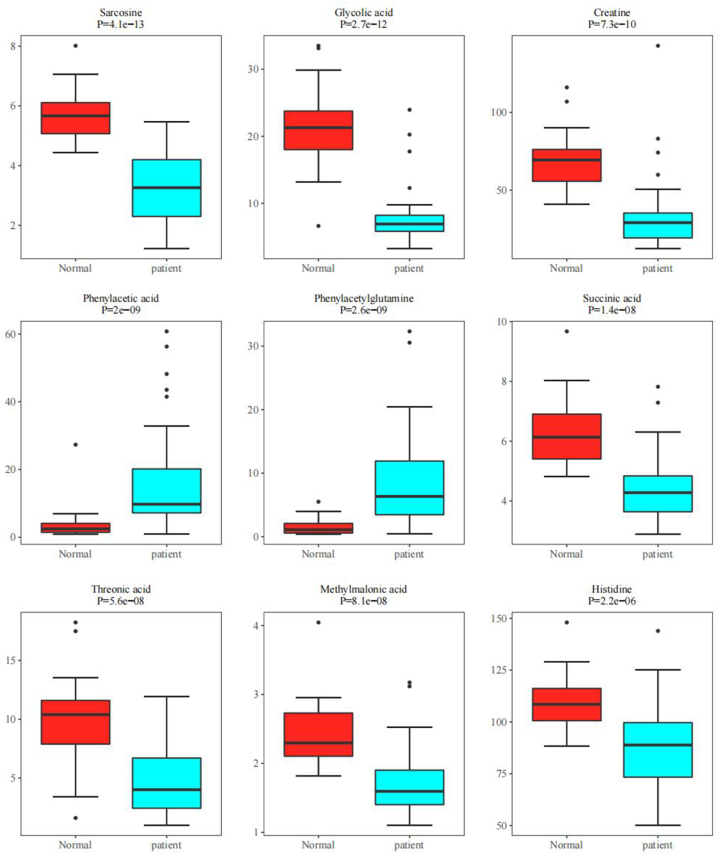
(A) ROC curve for the 10 characteristic diagnostic metabolites; (B) Evaluation of metabolite importance by Random Forest; (C–L) Box plots for the top 10 ranked unidimensional differential metabolites.

Through the random forest algorithm, 39 significant characteristic diagnostic metabolites were screened out (Fig. 3B). The top 10 metabolites include Sarcosine, Glycolic acid, Creatine, Phenylacetic acid, Phenylacetylglutamine, Succinic acid, Threonic acid, Methylmalonic acid, Histidine, and Imidazolepropionic acid. The area under the curve (AUC) of the ROC curve for the model using these 10 characteristic metabolites is equal to 1 (Fig. 3A). Among these AUC values, Sarcosine has the highest AUC of 0.949, while Imidazolepropionic acid has the lowest AUC of 0.795 (Table 2). Indicating high accuracy of the model. Glycolic acid, Creatine, and Sarcosine are the top three characteristic metabolites ranked by MeanDecreaseGini. These related components are closely related to the occurrence and development of gastric cancer. Except for Phenylacetic acid and Phenylacetylglutamine, all of them are expressed at lower levels in gastric cancer patients (Fig. 3C–L).

**Table 2:** Basic information and ROC curve analysis results of the 10 characteristic diagnostic metabolites.

	Metabolite	class	AUC	MeanDecreaseGini	CI1	CI1
1	Sarcosine	Amino Acids	0.949	3.675498931	0.901098103	0.996044754
2	Glycolic acid	Organic Acids	0.946	5.413611281	0.886586953	1
3	Creatine	Amino Acids	0.907	3.958991033	0.822227885	0.991105448
4	Phenylacetic acid	Benzenoids	0.898	2.447379694	0.812323993	0.983866483
5	Phenylacetylglutamine	Amino Acids	0.896	1.772019824	0.817094156	0.975286797
6	Succinic acid	Organic Acids	0.881	2.478942047	0.79367162	0.968233142
7	Threonic acid	Carbohydrates	0.868	1.309109285	0.774114708	0.961123387
8	Methylmalonic acid	Organic Acids	0.864	1.996615598	0.7690512	0.958567847
9	Histidine	Amino Acids	0.827	0.753045002	0.721323646	0.932009687
10	Imidazolepropionic acid	Imidazoles	0.795	0.711454578	0.684481196	0.905994994

#### 3.1.4 Differential metabolite enrichment analysis results

**Fig. 4:**
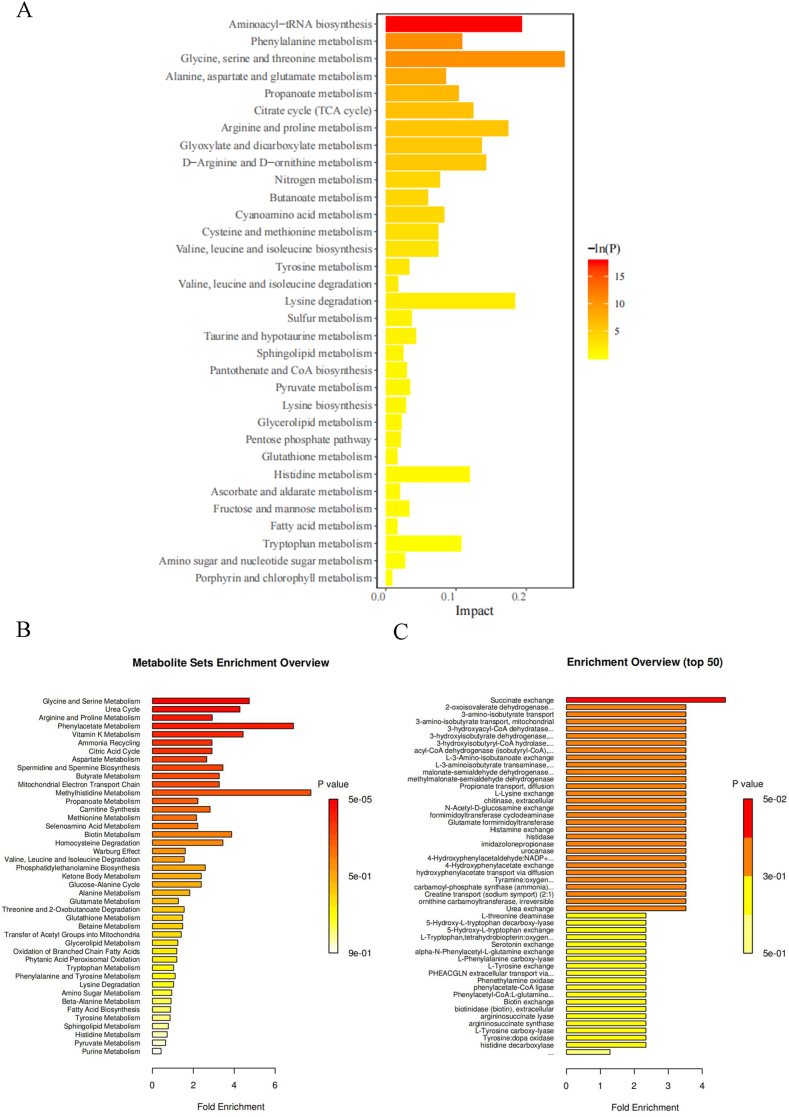
(A) Barplot of the top enriched pathways of metabolites in the KEGG database; (B) Pathway Enrichment Analysis Barplot Using Pathway-associated metabolite sets (Pathway-associated metabolite sets (SMPDB)); (C) Pathway Enrichment Analysis Barplot Using Pathway-associated metabolite sets (Predicted metabolite sets).

In the enrichment analysis bubble plot (Fig. 4A), each circle corresponds to a metabolic pathway. The x-axis represents the degree of pathway impact. The size of the circle is related to the pathway's impact value, with larger impact values corresponding to larger circles. The y-axis represents the negative logarithm of the P-value obtained from the pathway enrichment analysis. The color change is positively correlated with the negative logarithm of the P-value of the pathway change. The results show that the differential metabolites identified in the KEGG database were enriched in 42 different metabolic pathways (Fig. 4B and C), of which 13 pathways had a P-value less than 0.05. These pathways are: Aminoacyl-tRNA biosynthesis, Phenylalanine metabolism, Glycine, serine and threonine metabolism, Alanine, aspartate and glutamate metabolism (P < 0.001), Propanoate metabolism, Citrate cycle, Arginine and proline metabolism, Glyoxylate and dicarboxylate metabolism, D-Arginine and D-ornithine metabolism (P < 0.01), Nitrogen metabolism, Butanoate metabolism, Cyanoamino acid metabolism, and Cysteine and methionine metabolism (P < 0.05). The corresponding metabolites enriched in these 13 pathways are shown in Table 3. Among them, the differential metabolites are mainly enriched in three pathways: Aminoacyl-tRNA biosynthesis, Phenylalanine metabolism, and Glycine, serine and threonine metabolism.

**Table 3:** Metabolites enriched in the 13 pathways.

	Metabolic Pathway	Raw P	Enriched_Compounds	Impact
1	Aminoacyl-tRNA biosynthesis	0.000000024451	Asparagine、Histidine Arginine、Serine Methionine、Valine、Alanine Lysine、Threonine Tryptophan	0.19356
2	Phenylalanine metabolism	0.00002301	Phenylacetic acid Benzoic acid Phenylacetylglutamine Succinic acid、Fumaric acid p-Hydroxyphenylacetic acid	0.10909
3	Glycine, serine and threonine metabolism	0.000033668	Serine、Glyceric acid Sarcosine、Threonine Creatine、Tryptophan	0.25424
4	Alanine, aspartate and glutamate metabolism	0.00025673	Asparagine、Alanine Fumaric acid、Succinic acid	0.08571
5	Propanoate metabolism	0.0011373	Propionic acid Methylmalonic acid Succinic acid、Valine	0.10416
6	Citrate cycle (TCA cycle)	0.0022845	Succinic acid、Malic acid Fumaric acid	0.125
7	Arginine and proline metabolism	0.0034583	Ornithine、Arginine Creatine、Fumaric acid Sarcosine	0.17476
8	Glyoxylate and dicarboxylate metabolism	0.0043267	Glycolic acid、Glyceric acid Malic acid、Succinic acid	0.13637
9	D-Arginine and D-ornithine metabolism	0.0048485	Arginine、Ornithine	0.14286
10	Nitrogen metabolism	0.015341	Tryptophan、Asparagine Histidine	0.07692
11	Butanoate metabolism	0.016431	Butyric acid、Succinic acid Fumaric acid	0.06
12	Cyanoamino acid metabolism	0.019403	Asparagine、Serine	0.08333
13	Cysteine and methionine metabolism	0.039754	Serine、Methionine、Alanine	0.07408

## 4 16S rDNA sequencing

The intestine is the largest habitat for microbiota in the human body and serves as an essential "organ" in the host's immune and metabolic systems [17]. Increasing evidence suggests that intestinal microbiota imbalance may affect the occurrence and progression of systemic diseases beyond the intestine, and even influence the effectiveness of immunotherapy [18].We use fecal samples to study the composition of intestinal microbiota, which is convenient and non-invasive.

### 4.1 The intestinal microbial community structure at the phylum level

**Fig. 5:**
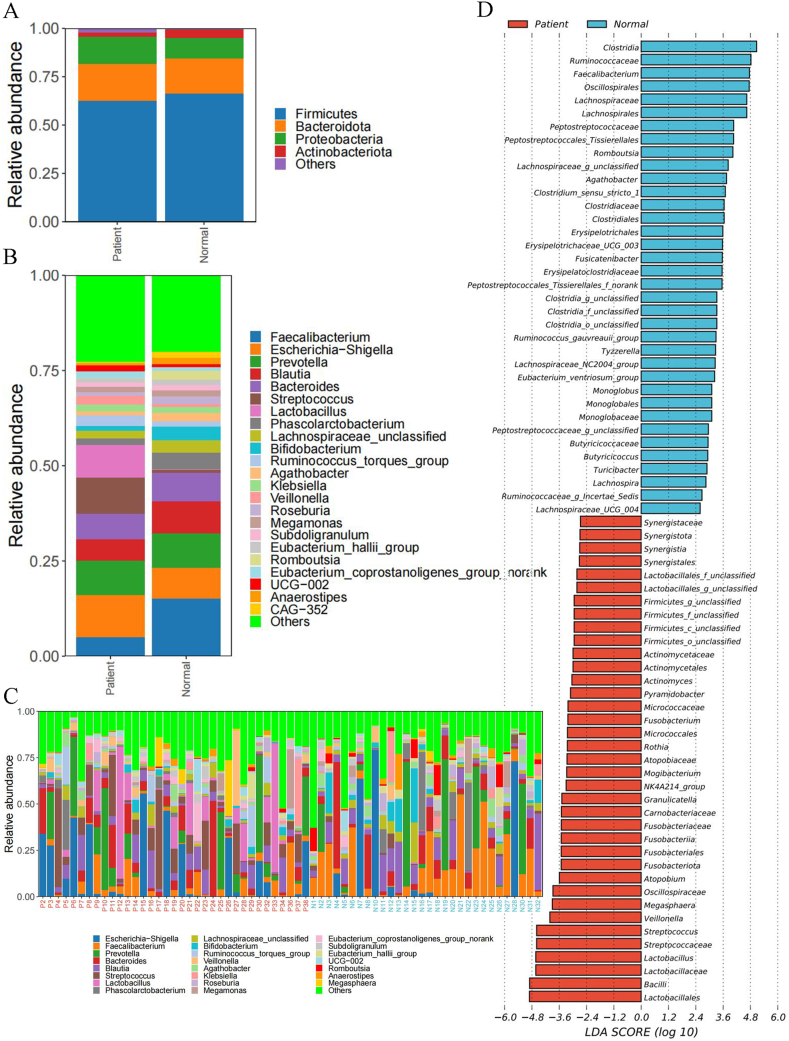
(A) Composition of the microbial community structure at the phylum level; (B) Composition of the microbial community structure at the genus level; (C) Composition of the microbial community structure in each sample; (D) Bar plot of LEfSe analysis.

At the phylum level, the relative abundance of intestinal microbiota is mainly composed of Actinobacteria, Proteobacteria, Bacteroidetes, and Firmicutes. Compared to the healthy group, patients with gastric cancer have a decreased expression of Firmicutes and Actinobacteria, while Bacteroidetes and Proteobacteria show an increased expression. Other bacterial groups also have higher contents and abundances (Fig. 5A).

### 4.2 Intestinal microbial community structure at the genus level

At the genus level, the intestinal microbiota is more diverse. The microbiota related to gastric cancer includes Lactobacillus, Streptococcus, and *Escherichia coli*. There expressions in the gastric cancer group are significantly higher than those in the healthy group. These genera dominate the intestinal microbiota in gastric cancer patients. In contrast, the expressions of Faecalibacterium, Phascolarctobacterium, and Bifidobacterium are significantly decreased (Fig. 5B).

To determine the microbiota types associated with advanced gastric cancer, we analyzed the microbiota using LEfSe and presented the results at the genus level (Fig. 5C). Compared to the healthy group, 73 microbial genera showed significant differences between the gastric cancer group and the healthy group. Among them, 36 were enriched in the healthy group samples, while 37 were enriched in the gastric cancer group samples. In gastric cancer, Lactobacillus, Lactococcus, Bacillus, Streptococcus, Veillonella, and Megasphaera were the most enriched genera. While Clostridium, Faecalibacterium, and Romboutsia were more enriched in the healthy group samples. These significant changes in the microbial community structure in gastric cancer are associated with an increased risk of gastric cancer and may serve as potential diagnostic biomarkers for gastric cancer. They deserve our close attention.

## 5 Correlation between differential plasma metabolites and differential fecal microbiota metabolites

**Fig. 6:**
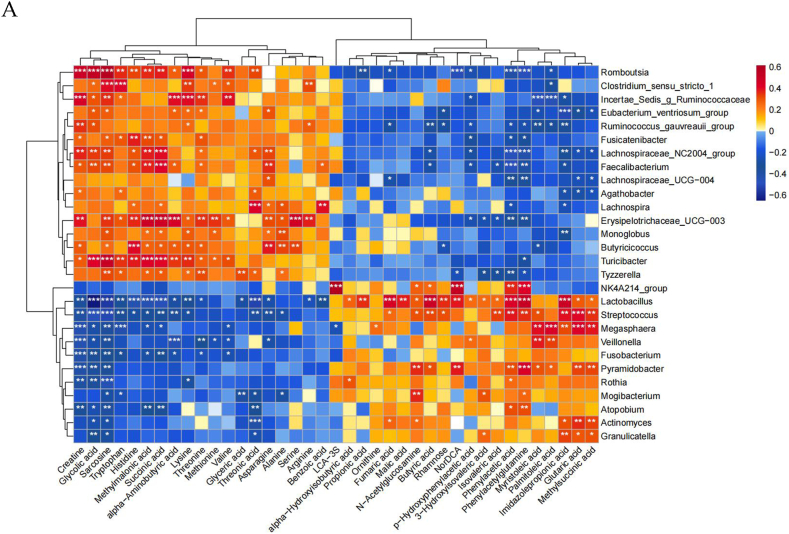
(A) Heatmap of joint analysis of differential plasma metabolomic profiles and gut microbiota profiles.

To further investigate the relationship between plasma metabolites and intestinal microbiota, we conducted a correlation analysis. Among the top ten differential metabolites, three plasma amino acids (creatine, sarcosine, and histidine), two organic acids (glycolic acid and succinic acid), and a carbohydrate (threonine) showed a negative correlation with Lactobacillus, Streptococcus, Veillonella, Megasphaera, and Actinobacteria. They showed a positive correlation with Faecalibacterium, Erysipelotrichaceae, Romboutsia, Ruminococcus, and Campylobacter. An organic acid (methylmalonic acid) and a plasma amino acid (phenylacetylglutamine), a phenyl derivative (phenylacetic acid), and an imidazole (imidazolpropionic acid) showed a positive correlation with Lactobacillus, Streptococcus, Veillonella, Megasphaera, and Actinobacteria. They showed a negative correlation with Faecalibacterium, Erysipelotrichaceae, Romboutsia, Ruminococcus, and Campylobacter (Fig. 6). Besides the top ten metabolites, we also found that Lysine, Tryptophan, NorDCA, Methionine, Asparagine, Arginine, and Serine were closely related to intestinal microbiota.

By comprehensively applying plasma metabolomics and 16S rDNA sequencing technology, we discovered significant changes in metabolic characteristics and intestinal microbiota structure in patients with advanced gastric cancer. There ia a significant correlation between the two. This not only helps us better understand the pathogenesis of gastric cancer but also provides new theoretical foundations for the diagnosis and treatment of gastric cancer.

## 6 Discussion

Gastric cancer is a common malignant tumor with high morbidity and mortality rates. Especially in the case of advanced gastric cancer, the lack of effective biomarkers makes it difficult to detect and diagnose in the early stages, and the underlying pathogenic mechanisms remain unclear. These limitations underscore the importance of gaining new insights into the pathogenesis of advanced gastric cancer. Modern medicine has delved into the genetic and epigenetic levels in understanding gastric cancer. This suggests that there may be significant differences in biological characteristics, clinical manifestations, treatment response, and prognosis. With the advent of gene sequencing, bioanalytical technologies, and big data analysis tools, the treatment of gastric cancer has entered the era of precision medicine.

In this study, we employed a combined approach of metabolomics and 16S rDNA sequencing to investigate the plasma metabolome and intestinal microbiota of 30 patients with stage IIIB-IV gastric cancer.

Through metabolomics analysis, we identified a total of 152 relevant plasma differential metabolites, of which 8 metabolites showed significant differences. These metabolites play crucial roles in biological processes such as protein synthesis, energy metabolism, and signal transduction, which are associated with the proliferation, migration, and other activities of gastric cancer.

In our study, several metabolites have been reported to be closely related to gastric cancer. For instance, alterations in fatty acid metabolism are associated with changes in the immune microenvironment of gastric cancer [19]. They are one of the most important hallmarks of cancer metastasis initiation [20]. Fatty acid-induced O-GlcNAcylation promotes the transcription and function of CD36 by directly modifying the S468 and T470 sites of CD36 through activation of the NF-κB pathway, thereby driving gastric cancer metastasis [21]. Among the fatty acids that have been extensively studied, docosahexaenoic acid has been confirmed by both in vitro and in vivo studies to induce apoptosis [22,23], inhibit cell invasion [24,26], and suppress cell growth [25] in gastric cancer cells. We identified fatty acid components such as Methylsuccinic acid, 3−Hydroxyisovaleric acid, Palmitoleic acid, and Myristoleic acid et al. But their mechanisms of action remain unclear and could be the focus of future research.

Methylmalonic acid (MMA) is one of the top 10 differential metabolites. It belongs to the category of organic acids. Elevated MMA is a good indicator of vitamin B12 deficiency, which is a common complication among patients after gastric resection. The study found that MMA levels were significantly increased in gastric cancer patients after gastric resection. These results suggest that additional assessment of MMA levels could help evaluate early changes in vitamin B12 levels in patients after gastrectomy and may even serve as a diagnostic marker for gastric cancer [27].

Amino acid are frequently found in tests as differential metabolitesand. It plays crucial roles in various biological processes such as protein synthesis, energy metabolism, and signal transduction. Current studies have indicated that L-arginine intervention promotes apoptosis. It can significantly increase the expression of apoptosis-promoting proteins p53, while significantly reduce the expression of anti-apoptotic proteins Bcl-2 and survivin [28,29]. Numerous studies have confirmed that arginine plays a vital role in immune function and intestinal mucosal protection. The combined use of arginine and glutamine in enteral and parenteral nutrition for patients after gastric cancer surgery has led to increased levels of albumin, serum protein, and transferrin, as well as decreased levels of C-reactive protein, C3, and C4 [30]. Cellular immune indicators were also significantly improved, and both apoptosis and PCNA expression in tumor tissues were improved [31,32]. Graziosi L et al. found that methionine can regulate the proliferation and migration of gastric tumor cells and reduce the production of peritoneal nodules [33]. These amino acids were all detected in our study and ranked high in terms of their importance.

Bile acids are conducive to the absorption of dietary fat and can also affect the process of gastric cancer [34]. Currently, the differential bile acid components we detected include NorDCA and LCA−3S. There are many studies on bile acids at present. Bile acid reflux can damage gastric mucosa [35], and high concentration of bile acids can induce apoptosis of gastric cancer cells [36]. Among them, hydrophobic bile acids such as chenodeoxycholic acid promote cell invasion through PKC activation and COX-2 induction in gastric cancer cells. Ursodeoxycholic acid weakens PGE2 synthesis by disturbing the bile acid pool, which affects tumor invasiveness [37], and induces cell death according to the intracellular signaling environment [38]. Secondary bile acids are typical carcinogenic bile acids [40,41]. For example, deoxycholic acid (DCA) has been shown to be related to alkaline gastritis [39]. It can promote the expression of E-cadherin and inhibit the invasion and migration of gastric cancer cells by upregulating the expression of MUC2 mRNA and downregulating the expression of Snail and MMP9 [42]. DCA can also induce apoptosis of gastric cancer cells through mitochondrial pathway, which is manifested by the increase of Bax/Bcl-2 ratio and the collapse of mitochondrial membrane potential [43].

Our research results show that glycolic acid, creatine and sarcosine are also closely related to the condition of gastric cancer. But we have read all the relevant literature, and found that there has been no detailed research on the mechanisms of these three metabolites in the literature. This provides us with new ideas for our future research. We believe that future research on the specific mechanisms of these metabolites will also be a new breakthrough point.

Our research findings are consistent with most of the previous studies. This indicate the reliability of our results. Furthermore, compared with previous reports, we have uncovered more changes in plasma metabolites related to advanced gastric cancer, which will be conducive to screening for new biomarkers.

In addition, metabolomics has also revealed the metabolic differences between gastric cancer cells and normal cells. Gastric cancer cells tend to have higher glycolytic activity and amino acid metabolic activity, which may be related to the rapid proliferation and increased energy demand of gastric cancer cells. Meanwhile, gastric cancer cells may also exhibit abnormal expression of certain specific metabolic pathways, which may be correlated with the malignancy, progression rate, and therapeutic response of gastric cancer.

Among the identified differential metabolites in the KEGG database, 42 differential metabolic pathways were enriched, with 13 pathways having a P-value less than 0.05. These metabolic pathways may ultimately lead to pathological changes. Further functional and clinical sample analysis of these metabolic pathways is needed to further prove their roles.

The study of metabolomics provides us with a new perspective and tool to gain a deeper understanding of the mechanisms of gastric cancer development. It offers new strategies and methods for the diagnosis, treatment, and prevention of gastric cancer. By further investigating the relationship between metabolites and gastric cancer, we can better discover the biological characteristics of gastric cancer.

The intestinal microbiota is the largest microecosystem in the human body. The ecological imbalance provides opportunities for the growth of pathogenic bacteria. The intestinal flora includes probiotics and conditional pathogens. The former mainly includes Lactobacillus and Bifidobacterium, while the latter includes Enterococcus, Escherichia, Shigella, Klebsiella, Streptococcus, and Streptococcus gastricus et al. [44].

Through 16SrDNA sequencing, we found that compared to the healthy group, the expression of Firmicutes and Actinobacteria was reduced in the gastric cancer group at the phylum level, while the expression of Bacteroidetes and Proteobacteria was increased. Bacteroidetes is an important clinical pathogen that contributes to anaerobic infections. It can break down polysaccharides in the intestine into short-chain fatty acids, increase the permeability of the intestinal mucosa, promote inflammation, and has a mutually reinforcing symbiotic relationship with Firmicutes. The imbalance in the ratio of Firmicutes to Bacteroidetes may indicate impaired intestinal absorption and inflammation, further promoting the occurrence of gastric cancer.

The bacterial flora in the gastric cancer group exhibited a more diverse composition at the genus level. The expression of Lactobacillus, Streptococcus, and *Escherichia coli* is significantly higher than in the healthy group. While the expression of Faecalibacterium, Phascolarctobacterium, and Bifidobacterium was significantly reduced. There were significant differences in 73 bacterial taxa between the two groups, with 37 taxa enriched in the gastric cancer group. We focused our attention on changes in Lactococcus, Lactobacillus, Bacillus, Streptococcus, Veillonella, and Megasphaera.

Lactobacillus has the closest relationship with gastric cancer. It can block the invasion and colonization of pathogens such as bacteria, viruses, and fungi through competition. It can also maintain the integrity of the epithelial barrier, and promote the production of antibacterial compounds [45,46]. However, more than 200 Lactobacillus-related infections have been found in immunocompromised individuals, such as cancer patients [47,48]. The increase in Lactobacillus abundance in gastric cancer is consistent with previous studies, showing a positive correlation between Lactobacillus content and disease severity in gastric cancer [49,50]. Lactobacillus proliferates in the stomach of gastric cancer patients and moves downward to the intestine [51]. Dynamic monitoring of Lactobacillus levels has important clinical significance for predicting the development of gastric cancer. In addition, some studies have found that Lactococcus and Megasphaera are related to gastric cancer [52], and the potential pathogenic bacterium Veillonella is significantly increased in gastric cancer [53,54,55]. This result is consistent with our findings. But the mechanism of action of Megasphaera and Veillonella is still unclear. Their relationship and mechanism of action with gastric cancer deserve further investigation.

Streptococcus is an anaerobic bacterium. Previous studies have also shown that the abundance of Streptococcus in gastric cancer tumor tissues has increased [56,57]. There have been research reports using Streptococcus as a diagnostic marker. Zhou et al. diagnosed gastric cancer based on Streptococcus anginosus and Streptococcus constellatus in fecal samples, with an AUC of 0.91 [58]. Yu et al. explored Streptococcus as a marker for liver metastasis of gastric cancer, with an AUC of 0.651 [59]. It is reported that the content of Streptococcus in colorectal cancer also increased significantly [60,61]. This indicate its potential as a diagnostic marker for gastrointestinal cancer. Our analysis also validates the above results.

Shili Liu et al. found that the number of beneficial bacteria such as Faecalibacterium, Roseburia, and Lachnospira decreased in gastric cancer. The number of Escherichia and Streptococcaceae increased in gastric cancer patients [62]. Previous studies have also reported significant changes in Roseburia and Escherichia among patients, which are related to the patient's disease status [63]. These findings are fully consistent with our results. This suggests that Roseburia, Escherichia, Faecalibacterium, and Lachnospira could be used as biomarkers to distinguish gastric cancer from health. This demonstrate the potential application value of gut microbiota in the diagnosis of gastric cancer.

The development of gastric cancer is associated with multiple risk factors. We conducted a correlation analysis of the components of these metabolic profiles and found that these differential metabolites are closely related to and mutually influence fecal microbiota.

We discovered that Lactobacillus, Faecalibacterium, and Bacteroides may collectively alter the abundance of fatty acyls and glycerophospholipid metabolites. The increased relative abundance of differential metabolites in pathways such as aminoacyl-tRNA biosynthesis and nucleotide sugar metabolism may be due to the collective influence of Lactobacillus, Streptococcus, Empedobacter, and Faecalibacterium. They may also jointly be responsible for carbohydrate synthesis. This indicates that these microbiota affect the degradation and synthesis of most metabolites, and the composition of different metabolites also affects the living environment of the microbiota.

These results indicate that there is a strong interaction between gastric cancer metabolites and intestinal microbiota, which jointly affect gastric cancer. For example, it has been reported that the aryl hydrocarbon receptor and *Helicobacter pylori* can interact with the tryptophan metabolite kynurenine to weaken the immune system, increase the production of inflammatory cytokines, and induce gastric cancer. In gastric macrophages, *Helicobacter pylori* inhibits the uptake of arginine, thus inhibiting the production of NO, reducing the killing of *Helicobacter pylori* and tumor angiogenesis [64]. A study by Noto JM et al. confirmed that *Helicobacter pylori* infection reduces the level of specific bile acids and the accumulation of secondary bile acids, inhibits the activation of harmful signaling pathways, and increases the occurrence of gastric cancer [65].

The homeostasis of amino acids and other compounds provides metabolic precursors for microbial growth. It also ensure the homeostasis of various metabolic products such as amino acids in the host. For example, in our research results, creatine and sarcosine are among the top three in importance. They participate in various pathways such as glycine, serine, and threonine metabolism, connecting key metabolic networks. Studies have shown that L-threonine can generate SCFAs, acetate, butyrate, and propionate. It plays an important role in maintaining normal intestinal function and integrity [66].

It is possible that the microbiota participates in the degradation and synthesis of most of the differential metabolites in these categories. Plasma metabolome and intestinal microbiota profiling have great potential in the detection and treatment of gastric cancer. They may help understand its underlying mechanisms. However, there are currently few studies on the specific mechanisms of their interactions, which is a major focus of future research on the impact of metabolome and microbiota profiling on gastric cancer.

The initial interpretation of these results suggests that differences in the microbiome and metabolome ultimately affect the occurrence and progression of gastric cancer. Many of the findings in this study are consistent with previously reported results, which confirms the reliability of our research results. In addition, our study reveals more plasma metabolites and changes in intestinal microbiota associated with advanced gastric cancer. The metabolome described in this study could serve as biomarkers for the diagnosis and treatment of gastric cancer or targets for its prevention. However, we must acknowledge some limitations in our study. First, the sample size is relatively small, and these potential new biomarkers need to be further validated in a larger number of patients and controls.

## 7 Conclusion

In summary, our study is the first to comprehensively apply plasma metabolomics and 16SrDNA sequencing technology. The experiments revealed the metabolic and intestinal microbiota characteristics of advanced gastric cancer as well as their correlations, and discovered more signature differential metabolites.These findings provide new perspectives and ideas for gastric cancer research, offer new potential biomarkers for the diagnosis and monitoring of advanced gastric cancer, and provide new evidence for the diagnosis, treatment, and prognosis of gastric cancer. In the future, we will further explore the application value of metabolomics and microbiota in gastric cancer research, aiming to make greater contributions to improving the survival rate and quality of life of patients with gastric cancer.

## CRediT authorship contribution statement

**Xinyi Feng:** Writing – original draft, Software, Resources, Project administration, Methodology. **Yu Zhang:** Formal analysis, Data curation. **Jun Feng:** Validation, Software. **Zhongjun Li:** Resources. **Zhi Zhang:** Resources, Methodology. **Lin Zhu:** Supervision, Methodology, Investigation. **Ruoyu Zhou:** Validation, Supervision. **Haibo Wang:** Writing – review & editing, Project administration, Conceptualization. **Xiaojun Dai:** Resources, Project administration. **Yanqing Liu:** Writing – review & editing, Validation, Project administration, Formal analysis.

## Ethics and consent declarations

Ethical approval have been obtained before the research study commenced or data was collected, and informed consent obtained before the collection of data from participants.

## For ethics approval

This study was reviewed and approved by Medical Ethics Committee of Yangzhou Traditional Chinese Medicine Hospital with the approval number:(2022) Ethics Review No. (31); dated:2022.11.04.

## For consent

All participants/patients (or their proxies/legal guardians) provided written informed consent to participate in the study and for their data to be published.

## Funding

National Natural Science Foundation of China(82104946, 82274603).

Natural Science Foundation of Jiangsu Province(BK20210817).

The Traditional Chinese Medicine Science and Technology Development Project of Jiangsu Province (Project code: QN202008, MS2022119).

The young scientific and technological talents uplift project of Jiangsu Association of Integrated Traditional Chinese and Western Medicine (JSZXTJ-2024-A05).

The Qing Lan Project of Yangzhou University.

The Postgraduate Research & Practice Innovation Program of Jiangsu Province (SJCX23_2047).

## Declaration of competing interest

The authors declare that the research was conducted in the absence of any commercial or financial relationships that could be construed as a potential conflict of interest.

## References

[1] Smyth E.C., Nilsson M., Grabsch H.I. (2020). Gastric cancer. Lancet.

[2] Sung H., Ferlay J., Siegel R.L. (2021). Global cancer statistics 2020: GLOBOCAN estimates of incidence and mortality worldwide for 36 cancers in 185 countries. CA A Cancer J. Clin..

[3] Cunningham D., Allum W.H., Stenning S.P. (2006). Perioperative chemotherapy versus surgery alone for resectable gastroesophageal cancer. N. Engl. J. Med..

[4] Davidson M., Okines A.F.C., Starling N. (2015). Current and future therapies for advanced gastric cancer. Clin. Colorectal Cancer.

[5] Gullo Irene, Carneiro Fatima, Oliveira Carla (2018). Heterogeneity in gastric cancer: from pure morphology to molecular classifications. Pathobiology.

[6] Martínez-Reyes I., Chandel N.S. (2021). Cancer metabolism: looking forward. Nat. Rev. Cancer.

[7] Liang Lingfan, Sun Fei, Wang Hongbo (2021). Metabolomics, metabolic flux analysis and cancer pharmacology. Pharmacol. Ther..

[8] Schmidt D.R., Patel R., Kirsch D.G. (2021). Metabolomics in cancer research and emerging applications in clinical oncology. CA A Cancer J. Clin..

[9] Nascentes Melo LM., Lesner N.P., Sabatier M. (2022). Emerging metabolomic tools to study cancer metastasis. Trends Cancer.

[10] Nicholson Jeremy K., Holmes Elaine, Lindon John C. (2004). The challenges of modeling mammalian biocomplexity. Nat. Biotechnol..

[11] Schirmer M., Smeekens S.P., Vlamakis H. (2016). Linking the human gut microbiome to inflammatory cytokine production capacity. Cell.

[12] Nardone G., Compare D. (2015). The human gastric microbiota: is it time to rethink the pathogenesis of stomach diseases?. United European Gastroenterol J.

[13] Lu Dongxue, Liu Feng, Yan Jin (2020). Research progress in TCM syndromes based on systems biology. Chin. J. Inf. Tradit. Chin. Med..

[14] Zhu Jingjing, Song Xiaoqing, Zhang Junbo (2018). Development of amino Acid⁃Based Ra⁃diopharmaceuticals for tumor imaging. Mini Rev. Med. Chem..

[15] Jing Fangyu, Hu Xin, Cao Yufeng (2018). Discriminating gastric cancer and gastric ulcer using human plasma amino acid metabolic profile. IUBMB Life.

[16] He Nana, Hu Lan, Chen Yin (2016). Effect of Yiqifusheng recipe on metabonomics of spleen-qi deficiency Syndrome in mice with gastric cancer. Journal of Xinjiang Medical University.

[17] Gu Yu, Liu Can, Zheng Ningning (2019). Metabolic and gut microbial characterization of obesity-prone mice under a high-fat diet. J. Proteome Res..

[18] McQuade J.L., Daniel C.R., Helmink B.A. (2019). Modulating the microbiome to improve therapeutic response in cancer. Lancet Oncol..

[19] Yang Shifei, Sun Boshi, Li Weijing (2022). Fatty acid metabolism is related to the immune microenvironment changes of gastric cancer and RGS2 is a new tumor biomarker. Front. Immunol..

[20] Beloribi-Djefaflia S., Vasseur S., Guillaumond F. (2016). Lipid metabolic reprogramming in cancer cells. Oncogenesis.

[21] Jiang Mingzuo, Hu Nan, Xu Bing (2019). Fatty acid-induced CD36 expression via O-GlcNAcylation drives gastric cancer metastasis. Theranostics.

[22] Lee S.E., Lim J.W., Kim H. (2009). Activator protein-1 mediates docosahexaenoic acid induced apoptosis of human gastric cancer cells. Ann. N. Y. Acad. Sci..

[23] Ortega L., Lobos-González L., Reyna-Jeldes M. (2021). The Ω-3 fatty acid docosahexaenoic acid selectively induces apoptosis in tumor-derived cells and suppress tumor growth in gastric cancer. Eur. J. Pharmacol..

[24] Wu M.H., Tsai Y.T., Hua K.T. (2012). Eicosapentaenoic acid and docosahexaenoic acid inhibit macrophage-induced gastric cancer cell migration by attenuating the expression of matrix metalloproteinase 10. J. Nutr. Biochem..

[25] Wu Quan, Yu Jianchun, Liu Yuqin (2010). Effect of combination acid and fluorouracil on human gastric carcinoma cell strain MGC803. Acta Acad. Med. Sin..

[26] Shekari N., Javadian M., Ghasemi M. (2020). Synergistic beneficial effect of docosahexaenoic acid (DHA) and docetaxel on the expression level of matrix metalloproteinase-2 (MMP-2) and MicroRNA-106b in gastric cancer. J. Gastrointest. Cancer.

[27] Lee S.M., Oh J., Chun M.R. (2019). Methylmalonic acid and homocysteine as indicators of vitamin B12 deficiency in patients with gastric cancer after gastrectomy. Nutrients.

[28] Kang Kai, Yu Tingting, Shu Xiaoliang (2014). The regulation of L-arginine on apoptosis in human gastric cancer cell line SGC-7901. Parenteral & Enteral Nutrition.

[29] Shu Xiaoliang, Kang Kai, Zhang Yingqiong (2013).

[30] Chen Liuzhen, Wang Zaiguo, Zheng Jinglei (2020). Effect of arginine combined with glutamine parenteral nutrition on postoperative nutrition and stress of gastric cancer patients. Smart Healthcare.

[31] Xia Chunxian, Kang Jian, Yang Yilian (2001). Effect of arginine and glutamine on the immune function and tumor cell proliferating activity in patient with advanced gastric cancer. Chin. J. Clin. Med..

[32] Zhou Dianwei, Yu Gang (2020). The effect of early immunomodulatory enteral parenteral nutrition on postoperative recovery in elderly patients with advanced gastric cancer. Chinese Journal of Gerontology.

[33] Graziosi L., Mencarelli A., Renga B. (2013). Epigenetic modulation by methionine deficiency attenuates the potential for gastric cancer cell dissemination. J. Gastrointest. Surg..

[34] Cronin J., Williams L., McAdam E. (2010). The role of secondary bile acids in neoplastic development in the oesophagus. Biochem. Soc. Trans..

[35] Redlak M.J., Power J.J., Miller T.A. (2008). Prevention of deoxycholate-induced gastric apoptosis by aspirin: roles of NF-kappaB and PKC signaling. J. Surg. Res..

[36] Jenkins G.J., Cronin J., Alhamdani A. (2008). The bile acid deoxycholic acid has a non-linear dose response for DNA damage and possibly NF-kappaB activation in oesophageal cells, with a mechanism of action involving ROS. Mutagenesis.

[37] Wu Yu-Chung, Chiu Chang-Fang, Hsueh Chung-Tzu (2018). The role of bile acids in cellular invasiveness of gastric cancer. Cancer Cell Int..

[38] Lim S.C., Han S.I. (2015). Ursodeoxycholic acid effectively kills drug-resistant gastric cancer cells through induction of autophagic death. Oncol. Rep..

[39] Goldner F.H., Boyce H.W. (1976). Relationship of bile in the stomach to gastritis. Gastrointest. Endosc..

[40] Noto J.M., Piazuelo M.B., Shah S.C. (2022). Iron deficiency linked to altered bile acid metabolism promotes Helicobacter pylori-induced inflammation-driven gastric carcinogenesis. J. Clin. Invest..

[41] Lavelle A., Nancey S., Reimund J.M. (2022). Fecal microbiota and bile acids in IBD patients undergoing screening for colorectal cancer. Gut Microb..

[42] Pyo J.S., Ko Y.S., Kang G. (2015). Bile acid induces MUC2 expression and inhibits tumor invasion in gastric carcinomas. J. Cancer Res. Clin. Oncol..

[43] Song Wei, Yang Haibo, Chen Pu (2013). Apoptosis of human gastric carcinoma SGC-7901 induced by deoxycholic acid via the mitochondrial-dependent pathway. Appl. Biochem. Biotechnol..

[44] Wang Tingting, Cai Guoxiang, Qiu Yunping (2012). Structural segregation of gut microbiota between colorectal cancer patients and healthy volunteers. ISME J..

[45] Chew S.Y., Cheah Y.K., Seow H.F. (2015). Probiotic Lactobacillus rhamnosus GR-1 and Lactobacillus reuteri RC-14 exhibit strong antifungal effects against vulvovaginal candidiasis-causing Candida glabrata isolates. J. Appl. Microbiol..

[46] Kozakova H., Schwarzer M., Tuckova L. (2016). Colonization of germ-free mice with a mixture of three lactobacillus strains enhances the integrity of gut mucosa and ameliorates allergic sensitization. Cell. Mol. Immunol..

[47] Boyle R.J., Robins-Browne R.M., Tang M.L. (2006). Probiotic use in clinical practice: what are the risks?. Am. J. Clin. Nutr..

[48] Cannon J.P., Lee T.A., Bolanos J.T. (2005). Pathogenic relevance of Lactobacillus: a retrospective review of over 200 cases. Eur. J. Clin. Microbiol. Infect. Dis..

[49] Coker O.O., Dai Z., Nie Y. (2018). Mucosal microbiome dysbiosis in gastric carcinogenesis. Gut.

[50] Ferreira R.M., Pereira-Marques J., Pinto-Ribeiro I. (2018). Gastric microbial community profiling reveals a dysbiotic cancer-associated microbiota. Gut.

[51] Eun C.S., Kim B.K., Han D.S. (2014). Differences in gastric mucosal microbiota profiling in patients with chronic gastritis, intestinal metaplasia, and gastric cancer using pyrosequencing methods. Helicobacter.

[52] Zhang Yangyang, Shen Jian, Shi Xinwei (2021). Gut microbiome analysis as a predictive marker for the gastric cancer patients. Appl. Microbiol. Biotechnol..

[53] Wang Zikai, Gao Xuefeng, Zeng Ranran (2020). Changes of the gastric mucosal microbiome associated with histological stages of gastric carcinogenesis. Front. Microbiol..

[54] Chen Changchang, Chen Linjie, Lin Lijun (2021). Research progress on gut microbiota in patients with gastric cancer, esophageal cancer, and small intestine cancer. Appl. Microbiol. Biotechnol..

[55] Liu Dehua, Zhang Luotong, Chen Si (2022). Analysis of gastric microbiome reveals three distinctive microbial communities associated with the occurrence of gastric cancer. BMC Microbiol..

[56] Liu Xiaosun, Shao Li, Liu Xia (2019). Alterations of gastric mucosal microbiota across different stomach microhabitats in a cohort of 276 patients with gastric cancer. EBioMedicine.

[57] Shao D., Vogtmann E., Liu A. (2019). Microbial characterization of esophageal squamous cell carcinoma and gastric cardia adenocarcinoma from a high-risk region of China. Cancer.

[58] Zhou Chengbei, Pan Siyuan, Jin Peng (2022). Fecal signatures of Streptococcus anginosus and Streptococcus constellatus for noninvasive screening and early warning of gastric cancer. Gastroenterology.

[59] Yu Dandan, Yang Jinru, Jin Min (2021). Fecal Streptococcus alteration is associated with gastric cancer occurrence and liver metastasis. mBio.

[60] Flemer B., Warren R.D., Barrett M.P. (2018). The oral microbiota in colorectal cancer is distinctive and predictive. Gut.

[61] Dinakaran V., Mandape S.N., Shuba K. (2019). Identification of specific oral and gut pathogens in full thickness colon of colitis patients: implications for colon motility. Front. Microbiol..

[62] Liu Shili, Dai Jianjian, Xiang Lan (2021). Intestinal bacteria are potential biomarkers and therapeutic targets for gastric cancer. Microb. Pathog..

[63] Qin Junjie, Li Yingrui, Cai Zhiming (2012). A metagenome-wide association study of gut microbiota in type 2 diabetes. Nature.

[64] Pirzadeh M., Khalili N., Rezaei N. (2022). The interplay between aryl hydrocarbon receptor, H. pylori, tryptophan, and arginine in the pathogenesis of gastric cancer. Int. Rev. Immunol..

[65] Noto J.M., Piazuelo M.B., Shah S.C. (2022). Iron deficiency linked to altered bile acid metabolism promotes Helicobacter pylori-induced inflammation-driven gastric carcinogenesis. J. Clin. Invest..

[66] Gaifem J., Gonçalves L.G., Dinis-Oliveira R.J. (2018 Sep 5). L-threonine supplementation during colitis onset delays disease recovery. Front. Physiol..

